# Gendered Perceptions of Odd and Even Numbers: An Implicit Association Study From Arabic Culture

**DOI:** 10.3389/fpsyg.2021.582769

**Published:** 2021-04-21

**Authors:** Timothy R. Jordan, Hajar Aman Key Yekani, Mercedes Sheen

**Affiliations:** ^1^Department of Psychology, Ibn Haldun University, Istanbul, Turkey; ^2^Department of Psychology, Heriot-Watt University, Dubai, United Arab Emirates

**Keywords:** numbers, gender, implicit associations, individualism, collectivism

## Abstract

Previous studies conducted in the United States indicate that people associate numbers with gender, such that odd numbers are more likely to be considered male and even numbers considered female. It has been argued that this number gendering phenomenon is acquired through social learning and conditioning, and that male-odd/female-even associations reflect a general, cross-cultural human consensus on gender roles relating to agency and communion. However, the incidence and pattern of number gendering in cultures outside the United States remains to be established. Against this background, the purpose of this study was to determine whether people from a culture and country very different from the United States (specifically, native Arabic citizens living in the Arabic culture of the United Arab Emirates) also associate numbers with gender, and, if they do, whether the pattern of these associations is the male-odd/female-even associations previously observed. To investigate this issue, we adopted the Implicit Association Test used frequently in previous research, where associations between numbers (odd and even) and gender (male and female faces) were examined using male and female Arabic participants native to, and resident in, the United Arab Emirates. The findings indicated that the association of numbers with gender does occur in Arabic culture. But while Arabic females associated odd numbers with male faces and even numbers with female faces (the pattern of previous findings in the United States), Arabic males showed the reversed pattern of gender associations, associating even numbers with male faces and odd numbers with female faces. These findings support the view that number gendering is indeed a cross-cultural phenomenon and show that the phenomenon occurs across very different countries and cultures. But the findings also suggest that the *pattern* with which numbers are associated with gender is not universal and, instead, reflects culture-specific views on gender roles which may change across cultures and gender. Further implications for understanding the association of numbers with gender across human societies are discussed.

## Introduction

Social conditioning develops early in life (e.g., Bussey and Bandura, 1999; Martin and Ruble, 2010; Martin and Slepian, 2018) and goes on to instill concepts of gender that influence substantially the ways in which humans perceive their social world. Indeed, social role theory (Eagly, 1987; Eagly and Wood, 2016; Eagly and Sczesny, 2019) suggests that, by observing the gender with which objects or actions are associated in everyday experiences, humans learn (and reinforce) which objects or actions in their environment are considered male (e.g., tools, fixing cars) or female (e.g., baking equipment, cooking). In this way, people associate everyday objects and actions with gender through conditioning and observational learning and, more abstractly, even create associations whereby cognitive representations of items share a conceptual similarity with prototypical representations of gender. It is well established, for example, that people associate otherwise “genderless” objects, such as food, typefaces, and furniture, with gender (e.g., Gal and Wilkie, 2010; Palumbo et al., 2015; Jordan et al., 2017). In particular, Gal and Wilkie (2010) found that food dishes are perceived as more masculine or more feminine depending on their menu descriptions, Jordan et al. (2017) found that typefaces constructed from thick strokes are perceived as more masculine than typefaces constructed from thin strokes, and Palumbo et al. (2015) found that abstract shapes are perceived as more feminine when they have curved edges rather than sharp.

But although the phenomenon of associating objects with gender is well-established, one special type of gender association in object perception concerns numbers. In a seminal study conducted with participants in the United States, Wilkie and Bodenhausen (2012; see also Wilkie and Bodenhausen, 2015) examined the effects of numbers on the perceived gender of ambiguous visual stimuli (actually, foreign names and babies’ faces) that were not readily identifiable by participants as either male or female. Remarkably, Wilkie and Bodenhausen (2012) found that these stimuli were assigned male and female characteristics depending on the numbers with which they were presented. Specifically, when presented with digits that were odd numbers, names and faces were more likely to be perceived as male, and when presented with digits that were even numbers, the same names and faces were more likely to be perceived as female.

In interpreting the results of their work, Wilkie and Bodenhausen (2012, 2015) argued that the association of numbers with gender they observed may reflect a general, cross-cultural consensus on gender roles relating to agency and communion. In particular, regarding the number 1 as a single independent figure (both numerically and visually, given the solitary shape of the digit) is consistent with widely-held stereotypically masculine qualities of agency and individualism, both of which are associated with people concerned primarily with themselves and their own achievements (see Hofstede and Bond, 1984; Triandis, 1989; Triandis and Gelfand, 1998). In contrast, the number 2 has a numerical value which suggests togetherness and cooperation, and a visually rounded appearance, all widely-held stereotypically feminine qualities consistent with relational themes of communion and collectivism, both of which are associated with people concerned primarily with the groups to which they belong and for which they care, in exchange for group loyalty (see Hofstede and Bond, 1984; Triandis, 1989; Triandis and Gelfand, 1998). These perceptions of an *individualistic 1* and a *collectivistic 2*, Wilkie and Bodenhausen (2012, 2015) argued, then influence people’s perceptions of other numbers within the same category (odd or even) and produce a widespread, cross-cultural tendency for humans to form the same pattern of male-odd and female-even associations between gender and numbers.

However, the incidence and pattern of number gendering across different countries and cultures remains to be established. In particular, empirical research into the association of numbers with gender conducted so far (Wilkie and Bodenhausen, 2012, 2015) has focused on the United States (sometimes known as a WEIRD society: Western, Educated, Industrialized, Rich and Democratic; for discussion, see Henrich et al., 2010) and more research is now needed to help develop these findings to provide a complete understanding of the extent and nature of the number gendering phenomenon across different cultures. A crucial step in this respect is to investigate the existence of the phenomenon in cultures outside the United States and, in particular, the existence in these cultures of the male-odd/female-even associations between gender and numbers reported by Wilkie and Bodenhausen (2012, 2015).

Of considerable relevance to this research is that gender stereotypes can vary greatly with the intersectionality between gender, nationalities, and cultures (Ridgeway et al., 1998; Ridgeway, 2001; Veenstra, 2011). Indeed, recent studies have demonstrated that the widely held stereotypes of “individualistic men” and “collectivistic women” are not held universally but may be influenced greatly by social and cultural values, and the “cultural moderation hypothesis” offers a useful framework for this effect (Cuddy et al., 2015). Over several studies, Cuddy et al. (2015) found that participants from the United States, a Western country that values individualism more highly than collectivism (Hofstede et al., 2010), rated men as more agentic and individualistic than women. On the other hand, participants from South Korea, a country that values collectivism more highly than individualism (Hofstede et al., 2010), rated men as more communal and collectivistic than women (Cuddy et al., 2015). These differences in the way that individualistic and collectivistic traits are assigned across different cultures suggest that men, rather than women, are more likely to claim (and be perceived as possessing) the attributes that are particularly valued in a given culture (Berger et al., 1972).

Given the potential impact of culture on gender stereotype, it seems wise to consider that the attribution of gender to odd and even numbers may be affected by the culture to which people belong. Indeed, in view of the influence of individualism and collectivism proposed by Wilkie and Bodenhausen (2012, 2015) on the number gendering phenomenon they observed, it may well be that the actual process of attributing gender to numbers observed so far in the United States (where individualism is valued more highly than collectivism) is not observed across all societies. But we can find only one example of a study of the attribution of gender to odd and even numbers conducted outside the United States; a brief online survey with participants from India (Wilkie and Bodenhausen, 2012). Here the findings were essentially the same as for research with United States participants, indicating that odd numbers were regarded as more masculine than even numbers, and so suggesting a cross-cultural generality of this pattern of effects. But India, unlike the United States, is a country that shows little distinction between its valuing of collectivism and individualism (Hofstede et al., 2010) and so the contribution of these findings to understanding the influences of these cultural values on the number gendering phenomenon is not clear. This situation is complicated further because the actual cultural orientations (individualism/collectivism) of the participants were not reported, and online surveys often provide little control over who actually takes part (see Hydock, 2017). Consequently, it is difficult to be sure what these findings with Indian participants actually indicate about the nature and generality of number gendering across different cultures, although the study certainly underscores the need for investigating the number gendering phenomenon in a culture outside the United States.

One culture which is very different from that of the United States (and other Western countries) is the dominant Arabic culture of the United Arab Emirates. Accordingly, the purpose of the present research was to cast new light on the generality of number gendering across human societies by investigating the existence of this phenomenon amongst Arabic citizens native to and living in the United Arab Emirates. The dominant Arabic culture of the United Arab Emirates differs substantially from the dominant culture of the United States (and other Western countries) and, according to research by Hofstede et al. (2010) on cross-cultural values, this difference occurs on a number of important national cultural dimensions. Of particular relevance for the present research is that the United Arab Emirates and the United States differ substantially on levels of individualism and collectivism, with the United Arab Emirates scoring a very low 25 for individualism (and a corresponding high level for collectivism) and the United States scoring a very high 91 for individualism (and a corresponding low level for collectivism; Hofstede et al., 2010). In view of these differences, and the potential role of collectivism and individualism in the number gendering phenomenon described by Wilkie and Bodenhausen (2012, 2015), the Arabic culture of the United Arab Emirates provided a valuable opportunity to extend previous work on the association of numbers with gender.

If the tendency for humans to associate numbers with gender has genuine cross-cultural generality, important support for this generality would be provided by evidence of this phenomenon with Arabic citizens native to and living in the Arabic culture of the United Arab Emirates. But, if this evidence were found, the *pattern* of associations between gender and numbers obtained would be especially informative about the *nature* of this generality across cultures. In particular, if the same pattern of male-odd/female-even associations found in previous research in the United States (and, indeed, with Indian participants; Wilkie and Bodenhausen, 2012, 2015) were also found in Arabic culture, this similarity would provide a compelling indication that the association of odd and even numbers with male and female gender, respectively, is itself a general characteristic of number gendering across cultures, with important implications for how humans generally associate numbers with gender.

But, in view of the distinct national differences in cultural values of individualism and collectivism that exist between the United States and the United Arab Emirates, and already identified by Hofstede et al. (2010), it remains to be seen if the pattern of male-odd/female-even associations found in previous research actually extends to Arabic culture. Of particular relevance here is that, because Arabic culture (unlike Western culture) is highly collectivistic, group identity and the concept of communion are particularly valued over the concept of agency and individualism (Hofstede et al., 2010). As a result, stereotypically masculine qualities of agency and individualism in Western culture may change in Arabic culture to encompass otherwise stereotypically feminine qualities of communion and collectivism. Indeed, this possibility is supported by the research which suggests that higher status groups in societies tend to be aligned with a culture’s core values (Cuddy et al., 2015; see also Tajfel, 1981; Fiske, 2010), and males in Arabic culture possess a far higher status than females, underscored by customs and practices in this culture (Zeffane, 2017). Men in Arabic culture, therefore, may claim (and be perceived as possessing) a collectivistic role as this attribute is especially valued in this culture (e.g., Berger et al., 1972). In a similar vein, when considering the lower status of females in Arabic culture, females may be more likely to be associated with individualistic roles as these are less valued.

Such a shift in cultural values, with males perceived as collectivistic members of society and females as individualistic, would have important implications for understanding the nature of the number gendering phenomenon. In particular, if the associations of male traits with odd numbers and female traits with even numbers reported by Wilkie and Bodenhausen (2012, 2015) have genuine cross-cultural generality, as Wilkie and Bodenhausen (2012, 2015) propose, this pattern of associations should also be observed in Arabic culture, despite substantive differences in cultural views concerning stereotypically masculine and feminine traits of individualism and collectivism, relative to those reported so far for Western culture. However, if the associations of numbers with gender reflect cultural perspectives on gendered attributions of individualism and collectivism, as Wilkie and Bodenhausen (2012, 2015) suggest, *but* these associations are *not* universal and can change with culture-specific views on gender roles, associations of numbers with gender in Arabic culture (if they are found) should be different from those reported so far for Western culture. Specifically, in contrast to the association of male traits with odd numbers and female traits with even numbers previously reported, we would expect Arabic culture to show evidence of a reversal of this pattern of gender associations with numbers, with male traits now being associated with even numbers and female traits with odd.

The rationale and design of the experiment we report followed the approach adopted by Wilkie and Bodenhausen (2012, 2015) and used the Implicit Association Test (IAT; Greenwald et al., 1998) to assess implicit associations between odd and even numbers and male and female gender. A major benefit of this approach is that the IAT offers a means of revealing associations that are automatic and less likely to be contaminated by the deliberate intentions or biases of participants, and so provides a clearer indication of the cognitive associations that really exist. To conduct the test, participants were presented with two types of visual stimulus, either digits (odd or even numbers) or adult human faces (male or female), each shown individually in the center of a screen. Adult faces were used to represent male and female because faces provide substantial natural cues to human gender and because the perception of facial gender appears to be especially effortless and automatic (e.g., Yan et al., 2017), both of which reduced the potential for task interference. To help in this process, all faces were also chosen to be unambiguously male or female. In addition, all faces were of Arabic appearance so as to be consistent with the purpose of the research which was to reveal gender associations made by Arabic citizens within Arabic culture. On each trial, participants were required to categorize accurately each number or face as quickly as possible using response options that paired face gender and number in different ways. In one response condition, response options were “male face or odd number” and “female face or even number” while in the other response condition, response options were “female face or odd number” and “male face or even number.” The logic of this approach in the IAT is that response combinations of faces and numbers that are more associated in memory (e.g., male faces and odd numbers; female faces and even numbers) should be easier to process than the reverse combinations (female faces and odd numbers; male faces and even numbers), and this processing difference should be revealed by faster reaction times for the more associated response combinations (see Greenwald et al., 1998, for further discussion).

## Materials and Methods

### Ethics Statement

This study was approved by the Research Ethics Committee of Zayed University, United Arab Emirates, and all participants provided written consent.

### Participants

Participants were 56 Arabic United Arab Emirates nationals (28 males), recruited using advertisements posted in the United Arab Emirates. All participants reported being native to and resident in the United Arab Emirates and these details were verified using official documentation and one-to-one interviews. All participants showed normal or corrected-to-normal visual ability using Bailey-Lovie (Bailey and Lovie, 1980) assessments (see also Jordan et al., 2011).

Participants’ responses to the DUREL assessment of religious involvement (Koenig and Büssing, 2010) indicated high levels of religiosity (out of a maximum of five) for both males (*M* = 4.48, *SD* = 0.47) and females (*M* = 4.44, *SD* = 0.48), with no significant difference (*t* = 0.27, *df* = 54, *p* = 0.79, two-tailed).

Participants also completed the 16-item individualism/collectivism scale to measure their own cultural orientation (Triandis and Gelfand, 1998). A mixed factors ANOVA for gender (male, female) and cultural orientation (collectivism, individualism) showed a main effect of cultural orientation, *F*(1, 54) = 1,090.80, *p* < 0.0001, *ƞ^2^* = 0.95, due to higher levels of collectivism (*M* = 5.73, *SD* = 0.42) than individualism (*M* = 2.27, *SD* = 0.77), which is consistent with the national pattern for the United Arab Emirates (Hofstede et al., 2010). Indeed, all participants showed higher scores for collectivism (range 5.00–6.00) than for individualism (range 1.00–3.44). No main effect of gender, *F*(1, 54) = 0.34, *p* = 0.56, *ƞ^2^* = 0.01, or interaction between gender and cultural orientation, *F*(1, 54) = 0.44, *p* = 0.51, *ƞ^2^* = 0.01, was observed.

*A priori* estimates of sample sizes required for the design of this experiment were obtained using G*Power (Faul et al., 2007) for a statistical power of 0.95 at an alpha level of 0.05 and a *ηp^2^* of 0.50. This power analysis indicated that a sufficient sample would be 54 participants, and so our sample of 56 was appropriately powered.

### Stimuli

**Figure 1:**
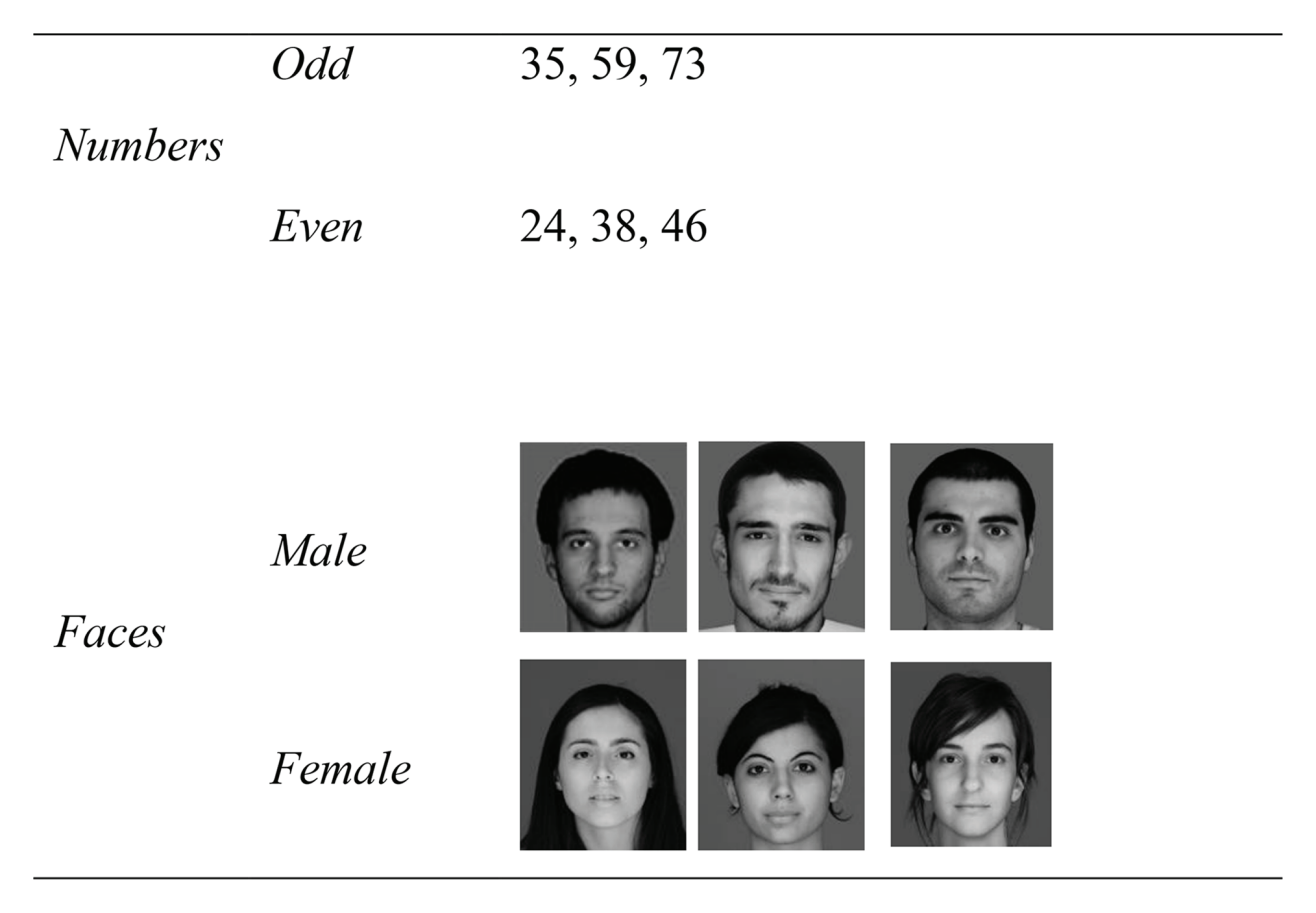
Examples of the stimuli used.

The IAT presented numbers (odd or even) and human faces (male or female) individually on a screen. A preliminary study showed that all faces were perceived unambiguously as their actual gender (male or female). The numbers used the same form of Arabic numerals (0–9) as in previous research (Wilkie and Bodenhausen, 2012, 2015) and this form is common in the United Arab Emirates. Following the logic of Wilkie and Bodenhausen (2012, 2015), faces specified a gender (male, female) and numbers provided a test of implicit gender association for these ostensibly gender-neutral stimuli. Face stimuli were provided by 16 faces, eight male and eight female, and number stimuli were provided by 16, 2-digit numbers, eight odd and eight even, each composed of either two odd or two even digits (see Figure 1). All face and number stimuli were shown to all participants, in a different random order for each participant. Practice items (six numbers, six faces) were shown at the start of each session to acquaint participants with the experimental procedure.

### Apparatus and Design

The experiment was run on an Apple Macintosh 3.7 GHz computer using Experiment Builder software, and stimuli were presented on a 27-in High Definition display screen. Each stimulus (number or face) was selected randomly and presented in the middle of the screen and participants categorized the number or face presented on each trial using two keys interfaced with the computer. Responses took place in two response association conditions. In one condition, participants used one key to respond “male face or odd number” and the other key to respond “female face or even number.” In the other condition, these pairings were altered so that participants used one key to respond “female face or odd number” and the other key to respond “male face or even number.” In this way, pairings of numbers and faces that are more cognitively associated should be easier to process and this should be revealed by faster reaction times to the more associated combinations. On each trial, the response options (male face or odd number/female face or even number OR female face or odd number/male face or even number) were indicated by labels in the left and right upper corners of the screen, corresponding to the left and right positions of two keys on an SR response box interfaced with the computer and placed in front of each participant. For each response association condition, response options were counterbalanced across screen locations and the two response keys. Stimulus presentations were synchronized with the refresh cycle of the display screen and participants made their responses *via* the SR response box which provided millisecond response-timing accuracy.

### Procedure

Participants took part individually in a sound-attenuated room and sat 60 cm from the display screen. Each stimulus (number or face) was presented one at a time in the center of the display and participants categorized the number or face presented on each trial. Participants were informed that their reaction time was of utmost importance and that they should respond as quickly as possible whilst trying to avoid making mistakes. When making a response, participants pressed one of the two keys corresponding to the two response options shown in the upper left and upper right corners of the screen.

## Results

**Figure 2:**
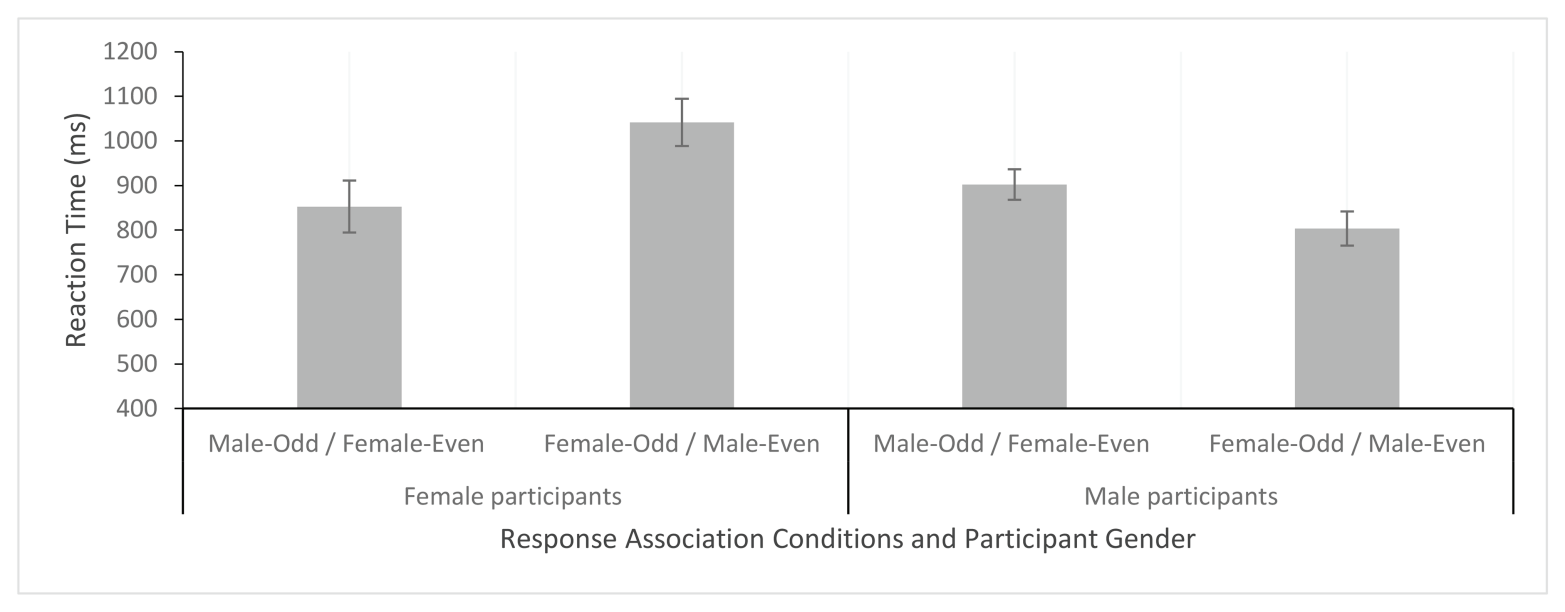
Mean reaction times (with standard error bars) for each response association condition (male-odd/female-even, female-odd/male-even) and participant gender.

In scoring the IAT, the strength of association was measured by comparing reaction times to different face-number pairings, with faster reaction times indicating a stronger association for those pairings. For example, if odd numbers are associated with males and even numbers associated with females, male face/odd number and female face/even number response options should produce faster reaction times. Mean reaction times for each participant gender (male, female) × response association condition (male face-odd number/female face-even number, female face-odd number/male face-even number) are presented in Figure 2. A mixed design ANOVA, with factors participant gender and response association condition, showed no main effect of participant gender, *F*(1, 54) = 2.40, *p* < 0.13, $\eta_{p}^{2}$ = 0.04, or response association condition, *F*(1, 54) = 2.85, *p* < 0.10, $\eta_{p}^{2}$ = 0.05. However, an interaction between participant gender and response association condition was found, *F*(1, 54) = 28.63, *p* < 0.0001, $\eta_{p}^{2}$ = 0.35. Analyses of simple main effects revealed a different pattern of reaction times for male and female participants. For female participants, reaction times were faster for male face-odd number/female face-even number than for female face-odd number/male face-even number association conditions, *F*(1, 54) = 24.78, *p* < 0.0001, $\eta_{p}^{2}$ = 0.32. However, this pattern was reversed for male participants, who were faster for female face-odd number/male face-even number than for male face-odd number/female face-even number association conditions, *F*(1, 54) = 6.71, *p* < 0.0001, $\eta_{p}^{2}$ = 0.11. Thus, important differences were revealed in the ways in which males and females associated gender with numbers.

As anticipated, each participant scored high in collectivism (see *Participants* section) and so fulfilled the expectations and requirements of conducting this experiment in the Arabic culture of the United Arab Emirates. As a result, it seemed unlikely that there would be enough variation to show any more detailed effect on the relationship between collectivism and IAT scores but, nevertheless, we examined this possibility. A Shapiro-Wilk test of distribution normality for collectivism ratings was significant for males *S-W* = 0.59, *df* = 28, *p* < 0.001 and females *S-W* = 0.64, *df* = 28, *p* < 0.001, reflecting a skew towards high collectivism scores. Accordingly, a Spearman correlation coefficient was used to examine the association between collectivism scores and IAT scores and showed no significant correlation, either for males (*r_s_* = −0.18, *p* = 0.35) or females (*r_s_* = 0.25, *p* = 0.21). A similar finding was observed for religiosity and IAT scores. A Shapiro-Wilk test of distribution normality for religiosity scores was significant for males *S-W* = 0.87, *df* = 28, *p* = 0.002 and females *S-W* = 0.80, *df* = 28, *p* < 0.001, reflecting a skew towards high religiosity scores, and a subsequent Spearman correlation coefficient showed no significant correlation between religiosity and IAT scores, either for males (*r_s_* = −0.07, *p* = 0.71) or females (*r_s_* = −0.25, *p* = 0.20).

Reaction times were also analyzed by the Greenwald et al. (2003) scoring algorithm. Instead of comparing within-person differences in raw latencies, these differences were standardized at the participant level by dividing differences between the mean reaction times of the two combined tasks by the *SD* of all reaction times in these tasks. The overall mean observed *d* score for reaction time was not significantly different from 0 whereas the overall mean *d* score for male and female participants was significantly different from 0. Moreover, there was a significant difference between *d* scores for male and female participants, *F*(1, 54) = 28.63, *p* < 0.0001, $\eta_{p}^{2}$ = 0.35.

## Discussion

The findings of this study provide clear indications that Arabic citizens native to and living in an Arabic country and culture associate numbers with gender. Indeed, both male and female Arabic citizens in this study showed the number gendering phenomenon. This evidence provides new insight into the incidence of number gendering across different societies and, when considered together with findings obtained previously in the United States, indicates that associating gender with otherwise genderless numbers occurs across very different countries and cultures. Therefore, although the extent to which number gendering exists across *all* countries and cultures remains to be established, these new findings add support to the view that number gendering is indeed a cross-cultural phenomenon, as Wilkie and Bodenhausen (2012, 2015) suggested.

But while indicating that number gendering exists across different cultures, these findings also suggest that the *pattern* of number gendering can vary with the culture and gender of the observer. In particular, whereas female Arabic participants in our study associated male faces with odd numbers and female faces with even numbers, and so showed the same general pattern seen previously in Western culture (Wilkie and Bodenhausen, 2012, 2015), male Arabic participants associated male faces with even numbers and female faces with odd numbers, in contrast to the previous findings. As a consequence, although this new evidence indicates that the association of male traits with odd numbers and female traits with even numbers reported previously by Wilkie and Bodenhausen (2012, 2015) has cross-cultural generality, this generality displays a greater complexity than previously considered.

Let us first turn to the findings observed with male participants. In line with the arguments and hypotheses described in the *Introduction*, Arabic culture is highly collectivistic, where group identity and the concept of communion are valued highly over the concept of agency which, in contrast, is valued highly in the individualistic cultures of the West (Hofstede and Bond, 1984; Triandis, 1989; Triandis and Gelfand, 1998; Hofstede et al., 2010). Indeed, a further indication of the dominant nature of collectivism in Arabic culture comes from the higher levels of collectivism reported by all participants in the present study (see *Participants*). But males in Arabic culture also possess a far higher status than females (Zeffane, 2017) and, as higher status groups tend to be aligned with a culture’s core values (Tajfel, 1981; Fiske, 2010; Cuddy et al., 2015), the association of even numbers with male traits made by Arabic males in this study is consistent with the dominant and highly valued role of collectivism in Arabic culture. In a similar vein, considering the lower status assigned to females in Arabic culture, especially by males, it would be consistent for Arabic males to associate Arabic females more with what Arabic males regard as culturally less-valued individualistic roles than with collectivistic roles, and so associate female traits more with odd numbers than even, and this is what was found.

These influential elements of Arabic culture explain why Arabic males in our study associated their own male gender with even numbers that represent the more highly valued concept of communion over the concept of agency. However, in contrast to male participants, female participants associated male traits with odd numbers and female traits with even numbers, and this pattern was not anticipated, not least because it suggests that females in Arabic culture hold attitudes towards gender that are similar to those held in Western culture (Wilkie and Bodenhausen, 2012, 2015) and yet are very different from those of their own Arabic male compatriots. At first glance, therefore, it may seem that Arabic females, like Arabic males, were adopting for themselves the highly valued role of collectivism in Arabic culture. But a closer inspection suggests a more plausible explanation.

Arabic females in the United Arab Emirates lead very different lives from Arabic males and, despite a recent modernizing agenda which has publicized women’s roles as leaders and entrepreneurs in society (e.g., Allagui and Al-Najjar, 2018), Arabic women in the United Arab Emirates grow up in a culture where explicit and implicit deference to males is widespread and the norm (Erogul and McCrohan, 2008). In this environment, therefore, it is unlikely that females associate themselves with what they perceive as higher-status traits, and the association of females with even numbers we observed for female participants is likely to reflect an alternative perception of collectivistic roles in Arabic society. The clear contender for this alternative perception is that, as in many countries and cultures, Arabic females regard themselves as collectivistic not because they have the same concept of collectivism as Arabic males but because females have powerful biological and social roles caring within the family unit (Al Jenaibi, 2015) and so associate collectivism with females in ways that are very different from those adopted by Arabic males. In a similar manner, as males have a higher status than females in Arabic culture, it would be consistent for Arabic females to associate Arabic males more with traits that exemplify status, power, and competence (Fiske et al., 2016), all of which are qualities typically associated with the concept of agency, and so associate Arabic male traits more with odd numbers than even. Accordingly, Arabic males and Arabic females may have very different perceptions of collectivism and of the values of collectivism and individualism in their lives, and so they each make different associations between genders and numbers. In this sense, the complex gender-based and gender-specific characteristics of Arabic culture offer a credible explanation of why Arabic females associate female traits with collectivistic even numbers and male traits with odd, while Arabic males claim the dominant and higher status collectivistic role that they perceive for themselves. Further research will help clarify how gender is assigned more generally to gender-neutral objects in Arabic culture, and, indeed, in other cultures. But the current findings provide an important indication of the influences that may be implicated in producing the association of gender with numbers in Arabic culture and, more generally, the complex pattern of influences that may inspire the number gendering phenomenon.

The contrasting associations made by Arabic males and Arabic females indicate that the association of numbers with gender can vary substantially with the gender of the individuals making the associations. In turn, this means that a full understanding of the number-gendering phenomenon requires understanding the role and effect of participants’ own genders, but this aspect of the association of gender with numbers is far from understood. The evidence provided by the original studies of Wilkie and Bodenhausen (2012, 2015) is not clear on this topic as the main focus of these studies was the existence of the number-gendering phenomenon itself rather than effects of participant gender. Nevertheless, Wilkie and Bodenhausen (2015) found that the male-odd/female-even pattern of gender associations reported first by Wilkie and Bodenhausen (2012) occurred for both male and female participants, although other findings suggested a rather mixed set of effects involving participant gender. The findings of the current study, however, demonstrate a clear, contrasting difference between the nature of the number-gendering phenomenon shown by males and females within the same culture, and more research will determine the full influence of these human-gender differences on number-gendering.

Following the findings of the present research, two concluding points should be made. The first is that evidence of number gendering across such very different countries and cultures as the United States and the United Arab Emirates implies a fundamental influence that is common to all human societies and which inspires the association of gender with abstract concepts of numbers that are patently removed from actually being male and female. There may be several contenders for this influence, but many theorists have noted that the binary presence of two biological sexes becomes apparent to humans soon after birth and affects greatly how humans categorize their social world (e.g., Bem, 1981; Simon and Gagnon, 1986; Eagly et al., 2000; Eagly and Karau, 2002; Eagly and Sczesny, 2019). Bem (1981), for example (see also Bem, 1993; Starr and Zurbriggen, 2017), proposed that humans develop gendered role expectations such that associations that differentiate between masculinity and femininity (e.g., anatomy, divisions of labor, personality attributes) are also used to form schemas which are then used to perceive and categorize other forms of information in their social world (Fiske and Taylor, 1984; Strack and Deutsch, 2004). Numbers are remarkably well-suited to a schema based on binary categorization because numbers are either odd or even (what mathematicians call *parity*), and so the perception of sexes and perception of numbers share a particularly powerful and readily observable construct. Indeed, as Wilkie and Bodenhausen (2015) point out, the binary category of odd and even has long been regarded as a core component of the cognitive representation of numbers (Shepard et al., 1975) and seems to develop as an important part of number representations during childhood (Berch et al., 1999). Accordingly, if the binary nature of sex is intrinsically, cognitively linked to the binary nature of odd and even numbers, and provides a cognitive basis for categorizing these numbers, it seems likely that the gendering of numbers will occur wherever genders and numbers coexist. As the binary nature of sex is common across all human societies, including the United States and the United Arab Emirates, the influence of this construct and its application to numbers would explain the presence of number gendering across these two very different countries and cultures. But the extent to which the gendering of numbers is driven by the visual or conceptual nature of numbers should not be presumed. Indeed, the association of odd and even numbers with categories of gender may reflect a powerful cognitive need to divide the social world into male and female categories where odd and even numbers provide the *best fit* for each category, rather than odd and even numbers providing the primary impetus for categorizing the visual or conceptual nature of each number as being inherently male or female. The findings of the present study add to this view by showing that the same numbers (odd or even) can be associated with *both* masculinity *and* femininity, depending on the gender of the human observer.

The second point is that, as research into the number gendering phenomenon develops, the paradigms and techniques used to reveal the processes underlying this aspect of human cognition will also develop and mature. The paradigm we used in this study (the IAT) and the technique of using faces to convey masculinity and femininity were chosen to be sensitive to the existence of gender associations and to be naturalistic and close to the environmental characteristics of the culture being studied. But it remains to be seen if other paradigms and techniques would produce similar effects. For example, determining facial gender is an especially automatic aspect of human perception (Yan et al., 2017), and was selected for this reason, but other more effortful aspects of gender perception (e.g., the explicit gendering of numbers) may often not have the same sensitivity, and so show different effects, or no effects at all (see also Wilkie and Bodenhausen, 2015).

In sum, the evidence reported here reveals that gendered associations with odd and even numbers exist in Arabic culture but that the nature of these associations shows important distinctions from that found previously in the United States. Since numbers and gender are ubiquitous parts of everyday life across all countries and cultures, the ways in which actual culture and actual gender influence abstract associations between numbers and gender have important and widespread implications for understanding social behavior. The current research provides new clues to how culture modulates the gendered ways in which males and females perceive numbers, and each other, but further research based on these findings will help develop our knowledge of the number gendering phenomenon and how it reflects the ways in which humans generally interact with their social world. Indeed, while the purpose of the present work was to address the role of collectivism and individualism in the number gendering phenomenon across cultures (Wilkie and Bodenhausen, 2012, 2015), other aspects of number perception require further investigation to provide a complete understanding of the ways in which numbers and gender integrate in human cognition. For example, even numbers have a greater familiarity in societies because they are more common (Lochy et al., 2000) and mathematical operations may be less difficult when they involve even numbers rather than odd (Knight and Behrens, 1928; Hines, 1990). Indeed, even numbers also appear to be processed more rapidly and more fluently than odd numbers (Hines, 1990), and number gendering might reflect links between the differential liking of even and odd numbers and the likeability of feminine stereotypes (Eagly and Mladinic, 1994). There seems little doubt that while numbers and gender are both plainly apparent in human societies, their interaction in producing the number gendering phenomenon is likely to be of considerable complexity.

## Data Availability Statement

The raw data supporting the conclusions of this article will be made available by the authors, without undue reservation.

## Ethics Statement

The studies involving human participants were reviewed and approved by Research Ethics Committee of Zayed University. The patients/participants provided their written informed consent to participate in this study. Written informed consent was obtained from the individual(s) for the publication of any potentially identifiable images or data included in this article.

## Author Contributions

TJ, HY, and MS conceived the study. HY analyzed the data. TJ and MS wrote the manuscript. All authors contributed to the article and approved the submitted version.

### Conflict of Interest

The authors declare that the research was conducted in the absence of any commercial or financial relationships that could be construed as a potential conflict of interest.
